# NPY mediates the rapid feeding and glucose metabolism regulatory functions of AgRP neurons

**DOI:** 10.1038/s41467-020-14291-3

**Published:** 2020-01-23

**Authors:** Linda Engström Ruud, Mafalda M. A. Pereira, Alain J. de Solis, Henning Fenselau, Jens C. Brüning

**Affiliations:** 1 Department of Neuronal Control of Metabolism, Max Planck Institute for Metabolism Research, Gleueler Strasse 50, 50931 Cologne, Germany; 2 Excellence Cluster on Cellular Stress Responses in Aging Associated Diseases (CECAD) and Center for Molecular Medicine Cologne (CMMC), University of Cologne, Joseph-Stelzmann-Strasse 26, 50931 Cologne, Germany; 3 Center for Endocrinology, Diabetes and Preventive Medicine (CEDP), University Hospital Cologne, Kerpener Strasse 26, 50924 Cologne, Germany; 4 Synaptic Transmission in Energy Homeostasis Group, Max Planck Institute for Metabolism Research, Gleueler Strasse 50, 50931 Cologne, Germany

**Keywords:** Feeding behaviour, Metabolism

## Abstract

Activation of Agouti-Related Peptide (AgRP)-expressing neurons promotes feeding and insulin resistance. Here, we examine the contribution of neuropeptide Y (NPY)-dependent signaling to the diverse physiological consequences of activating AgRP neurons. NPY-deficient mice fail to rapidly increase food intake during the first hour of either chemo- or optogenetic activation of AgRP neurons, while the delayed increase in feeding is comparable between control and NPY-deficient mice. Acutely stimulating AgRP neurons fails to induce systemic insulin resistance in NPY-deficient mice, while increased locomotor activity upon AgRP neuron stimulation in the absence of food remains unaffected in these animals. Selective re-expression of NPY in AgRP neurons attenuates the reduced feeding response and reverses the protection from insulin resistance upon optogenetic activation of AgRP neurons in NPY-deficient mice. Collectively, these experiments reveal a pivotal role of NPY-dependent signaling in mediating the rapid feeding inducing effect and the acute glucose regulatory function governed by AgRP neurons.

## Abstract (web summary)

AgRP-expressing neurons regulate feeding, glucose homeostasis and locomotor activity, but the neurotransmitters that mediate these effects are unclear. Here the authors show that neuropeptide Y in these neurons regulates rapid feeding responses and insulin sensitivity, but not locomotor activity.

## Introduction

Agouti-related peptide (AgRP) neurons integrate numerous signals communicating the energy state of the organism, such as leptin, insulin, and ghrelin^1^. In addition, it was more recently revealed, that they also transiently adapt their firing properties to sensory food perception^2^. Upon the integration of these energy-state-sensing signals they in turn adapt food intake. Accordingly, ablation of these cells in adult mice causes anorexia^3,4^, while optogenetic or chemogenetic stimulation of AgRP neurons evokes voracious feeding^5–7^. Importantly, state-dependent regulation of AgRP neuron activity not only acts to adapt feeding responses but also to coordinate multiple autonomic and behavioral responses in accordance to energy availability of the organism. This has been particularly exemplified for the CNS-dependent regulation of glucose homeostasis. Earlier work had shown that the systemic insulin resistance resulting from brain-wide deletion of the insulin receptor gene in mice can largely be phenocopied by selective inactivation of the insulin receptor in AgRP neurons^8,9^. Moreover, acute optogenetic or chemogenetic activation of AgRP neurons induces insulin resistance independent of its feeding-regulatory action^7^. These findings highlight the integrative metabolism regulatory role of AgRP neurons and assign them an important role not only in the development of obesity but also in the deregulation of glucose homeostasis as observed during the development of diabetes mellitus^1^.

Of note, in addition to their characteristic neuropeptide AgRP, AgRP neurons also release neuropeptide Y (NPY) as well as gamma-aminobutyric acid (GABA) to regulate downstream neurons, yet the importance of these neurotransmitters in regulating both feeding and insulin sensitivity remains unclear. Previous studies had indicated that the ability of chemogenetic AgRP neuron activation to promote feeding depends on both NPY and GABA release from these cells^10^. A recent study, however, demonstrated that while NPY release is necessary for the ability of optogenetic AgRP neuron activation to fully induce a rapid feeding response, the ability of chemogenetic AgRP neuron activation remained largely unaltered in the absence of NPY^11^. Nevertheless, these studies had not investigated, whether the insulin resistance regulatory role of AgRP neuron activation may depend on functional NPY expression in these neurons. Therefore, in the present study we have compared the ability of chemogenetic and optogenetic AgRP neuron activation to stimulate feeding, as well as to induce insulin resistance and to affect locomotor activity either in the presence or absence of NPY.

## Results

### Optogenetic stimulation of AgRP neurons in the absence of NPY

**Fig. 1:**
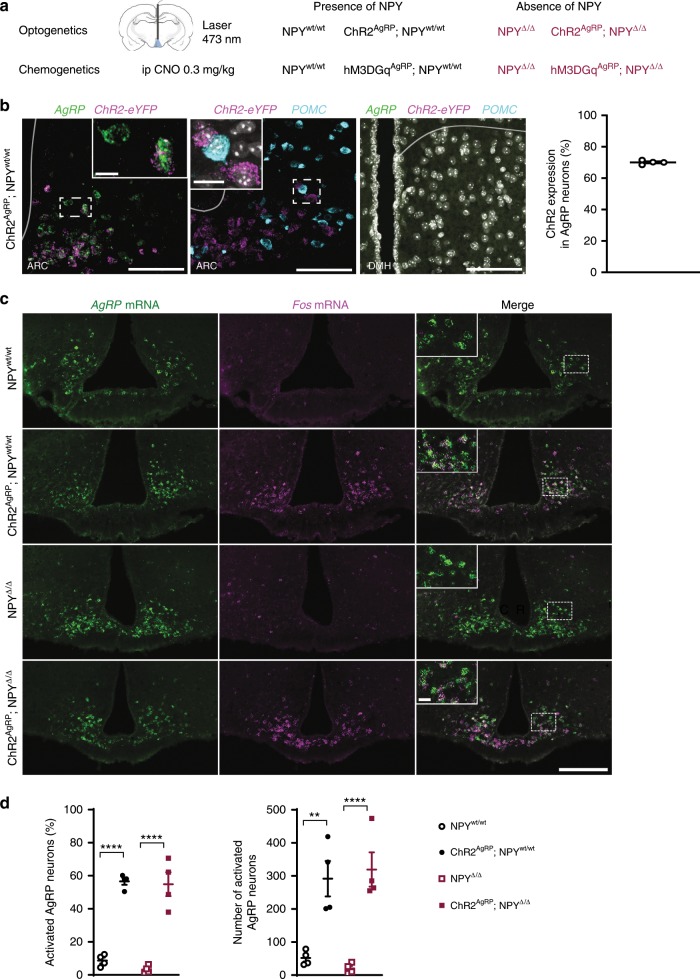
NPY-deficient mice retain AgRP neuronal activation. **a** Schematic representation of the optogenetic and chemogenetic strategies used to activate AgRP neurons in NPY-expressing and NPY-deficient mice. **b** Expression of *ChR2* occurs exclusively in AgRP neurons in the ARC. DAPI is depicted in gray in the DMH image (*n* = 4 mice). **c**, **d** Optogenetic activation of AgRP neurons occurs to the same extent in mice with and without NPY expression (*n* = 4 mice per group). Scale bar = 100 µm in **b**, 200 µm in **c**. Scale bars in insets = 20 µm. Data are shown as mean ± s.e.m. Statistical analysis is represented by **p ≤ 0.01 and ****p ≤ 0.0001 as determined by one-way ANOVA followed by Tukey post hoc test. Source data are provided as a Source Data file.

In order to investigate the contribution of NPY-dependent signaling to the diverse biological responses initiated by optogenetic stimulation of AgRP neurons, we crossed mice expressing Channelrhodopsin-2 (ChR2) from the ROSA26 locus in a Cre-dependent manner (ROSA26ChR2) with those heterozygous for a null mutation in the NPY locus (NPY^Δ/wt^-mice)^12,13^. Further intercrosses with AgRP-IRES-Cre mice^14^, yielded littermates, which were either controls (ROSA26ChR2^fl/wt^; AgRPCre^wt/wt^; NPY^wt/wt^, i.e. NPY^wt/wt^), which expressed ChR2 in AgRP neurons in the presence of NPY (ROSA26ChR2^fl/wt^; AgRPCre^Cre/wt^; NPY^wt/wt^, i.e. ChR2^AgRP^; NPY^wt/wt^), which lacked NPY expression in the absence of ChR2 expression in AgRP neurons (ROSA26ChR2^fl/wt^; AgRPCre^wt/wt^; NPY^Δ/Δ^, i.e. NPY^Δ/Δ^), or those expressing ChR2 in AgRP neurons and lacking NPY (ROSA26ChR2^fl/wt^; AgRPCre^Cre/wt^; NPY^Δ/Δ^, i.e. ChR2^AgRP^; NPY^Δ/Δ^) (Fig. 1a).

To first assess the specificity of AgRP neuron-restricted ChR2-expression in ChR2^AgRP^-mice, we performed an analysis of mRNA expression of eYFP, which is co-expressed with ChR2 in a Cre-dependent manner in these mice as well as an analysis of *AgRP* and *POMC* mRNA-expression (Fig. 1b). This analysis revealed that 70% of AgRP-expressing neurons in the ARC expressed ChR2, while ChR2-expression was not detectable in POMC-expressing neurons in the ARC, nor in neurons of the dorsal medial hypothalamus (DMH) (Fig. 1b).

Next, we aimed to define whether these animal models represent a valid approach to study the importance of NPY-dependent signaling independent from a possible alteration in ChR2-mediated AgRP neuron activation. Therefore, we compared the light-evoked activation of AgRP neurons in NPY-deficient and control mice. Activation of AgRP neurons was assessed by performing double in situ hybridization for *AgRP* and *Fos* mRNA after in vivo optogenetic stimulation (Fig. 1c, d, Supplementary Fig. 1). This analysis revealed that blue light (473 nm) laser illumination of the ARC similarly induced activation of AgRP neurons in both ChR2^AgRP^; NPY^wt/wt^ and ChR2^AgRP^; NPY^Δ/Δ^ mice (Fig. 1c, d). Thus, deficiency of NPY does not affect the ability of AgRP neurons to undergo ChR2-dependent activation upon laser illumination.

**Fig. 2:**
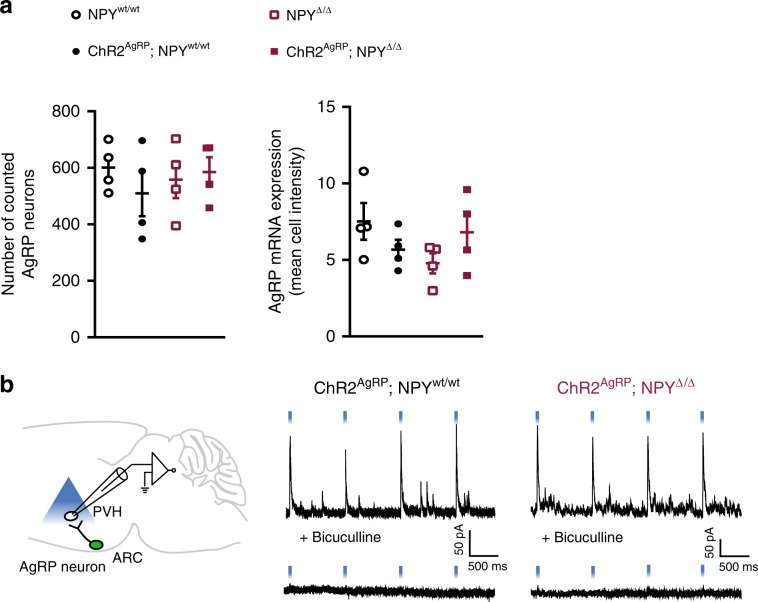
NPY-deficient mice retain *AgRP* expression and GABA release. **a** Graphs showing that *AgRP* mRNA levels, as determined by number of neurons (left) as well as mean cell intensity (right), do not differ between genotypes (*n* = 4 per group). **b** Schematic representation of the electrophysiological approach (left) and recording examples (right) of light-evoked GABAergic IPSCs in PVH neurons in ChR2^AgRP^; NPY^wt/wt^ and ChR2^AgRP^; NPY^Δ/Δ^ mice (*n* = 2 mice per group). Data are shown as mean ± s.e.m. Source data are provided as a Source Data file.

To investigate, whether the lack of NPY affects the expression of AgRP, we quantified both the number of *AgRP*-expressing cells and the mean cell intensity for *AgRP* expression in the different groups of animals. *AgRP* mRNA expression was similar in animals of the four genotypes as assessed by in situ hybridization (Figs. 1c and  2a). Thus, our mouse models allow us to define the effect of NPY deficiency in the presence of unaltered *AgRP* expression.

To further determine, whether NPY deficiency impairs GABA release from AgRP neurons upon optogenetic stimulation, we performed brain slice electrophysiology studies. To this end, we recorded light-evoked inhibitory postsynaptic currents (IPSCs) from unidentified neurons in the PVH in brain slices containing ChR2-expressing AgRP neuron terminals (Fig. 2b). Blue light illumination evoked IPSCs, which were completely blocked by the GABA-A receptor antagonist bicuculline, in PVH neurons from both ChR2^AgRP^; NPY^wt/wt^ and ChR2^AgRP^; NPY^Δ/Δ^ mice (Fig. 2b). Of interest, we failed to detect significant differences in the amplitudes of light-evoked IPSCs in our recordings from ChR2^AgRP^; NPY^wt/wt^(110.8 ± 88.51 pA) and ChR2^AgRP^; NPY^Δ/Δ^ mice (116.8 ± 65.13 pA). However, we found a slightly higher connectivity rate in mice lacking NPY expression (ChR2^AgRP^; NPY^wt/wt^ mice: 38%, ChR2^AgRP^; NPY^Δ/Δ^ mice: 56%), which is likely due to developmental compensations of GABAergic signaling in the absence of NPY signaling. These findings are consistent with a previous report describing an increased GABAergic synaptic transmission between AgRP neurons and PVH neurons in the absence of NPY expression^15^. Nevertheless, deficiency of NPY fails to overall affect the ability of AgRP neurons to inhibit downstream PVH neurons through GABA release. Together, these experiments show the successful establishment of an optogenetic mouse model that allows us to investigate the contribution of NPY-dependent signaling in response to AgRP neuron activation upon comparable ChR2-dependent cell activation, with unaltered *AgRP* expression and largely unaltered GABAergic signaling initiated by these neurons.

**Fig. 3:**
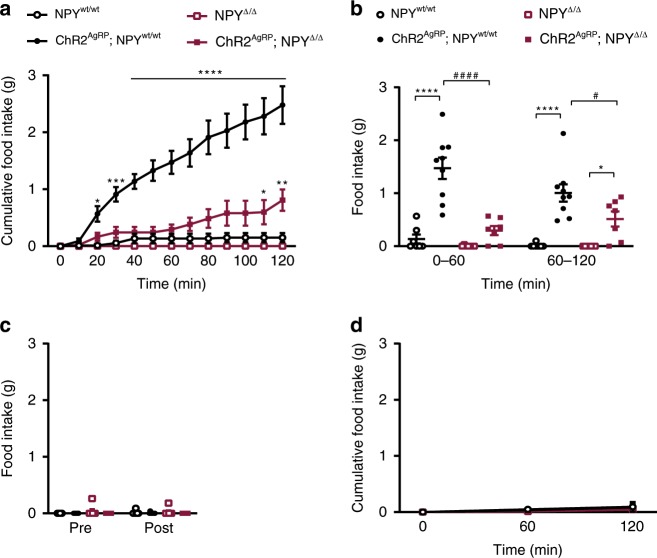
NPY is necessary for the acute feeding response upon optogenetic activation of AgRP neurons. **a**, **b** Cumulative and total food intake upon AgRP neuronal activation in the presence and in the absence of NPY (*n* = 7 mice for NPY^wt/wt^, NPY^Δ/Δ^ and ChR2^AgRP^; NPY^Δ/Δ^, and *n* = 9 mice for ChR2^AgRP^; NPY^wt/wt^). **c** Food intake one hour prior to light illumination of the ARC (pre) and one hour after lasers were turned off (post) is similar in all genotypes (*n* = 7 mice for NPY^wt/wt^, NPY^Δ/Δ^ and ChR2^AgRP^; NPY^Δ/Δ^, and *n* = 9 mice for ChR2^AgRP^; NPY^wt/wt^). **d** Cumulative food intake in the absence of light illumination (*n* = 4 mice for NPY^wt/wt^ and ChR2^AgRP^; NPY^Δ/Δ^, *n* = 3 mice ChR2^AgRP^; NPY^wt/wt^ and *n* = 5 mice for NPY^Δ/Δ^). Data are shown as mean ± s.e.m. Statistical analysis is represented by *p ≤ 0.05, ***p ≤ 0.001, ****p ≤ 0.0001, ^#^*p* ≤ 0.05, and ^####^*p* ≤ 0.0001 as determined by two-way ANOVA followed by Tukey post hoc test. # represents comparisons between NPY-expressing and NPY-deficient animals. Source data are provided as a Source Data file.

To investigate the effect of abrogated NPY signaling on the ability of AgRP neuron activation to stimulate food intake, we compared the feeding response upon light illumination of the ARC in the different groups of mice. As expected, in NPY^wt/wt^ and NPY^Δ/Δ^ mice, thus in the absence of ChR2 expression in AgRP neurons, light illumination of the ARC failed to increase light-cycle feeding (Fig. 3a, b). In ChR2^AgRP^; NPY^wt/wt^ mice, light illumination of the ARC for 2 h induced a rapid (within 20 min) and profound increase of feeding, while the same stimulation failed to affect food intake within the first 60 min in ChR2^AgRP^; NPY^Δ/Δ^ mice (Fig. 3a, b). However, after 60 min of light illumination, also ChR2^AgRP^; NPY^Δ/Δ^ mice responded with a steady increase in feeding, yet not reaching the same magnitude as compared with ChR2^AgRP^; NPY^wt/wt^ mice (Fig. 3a, b). Of note, feeding responses one hour prior to light illumination of the ARC (pre) as compared with 1 h after lasers were turned off (post) did not significantly differ between all four groups of animals (Fig. 3c). Moreover, daytime food intake over the same time of analysis in the absence of light-stimulation did not reveal any differences between mice of the different genotypes, confirming that the observed differences upon blue light illumination were the specific result of AgRP neuron activation in the presence or absence of NPY (Fig. 3d).

Since increased activity of AgRP neurons upon fasting critically contributes to the post-fast feeding response^16^, we next performed fasting/refeeding experiments in control and NPY-deficient mice. While the basal food intake over 24 h did not differ between control mice and NPY-deficient mice, NPY-deficient mice showed a significantly reduced food intake, as compared with control mice, in the first hour of refeeding after a 16 h fast (Supplementary Fig. 2a, b), when endogenous AgRP neuron activity is high. Thus, NPY signaling is partly responsible for the rapid endogenous food intake regulation after fasting.

**Fig. 4:**
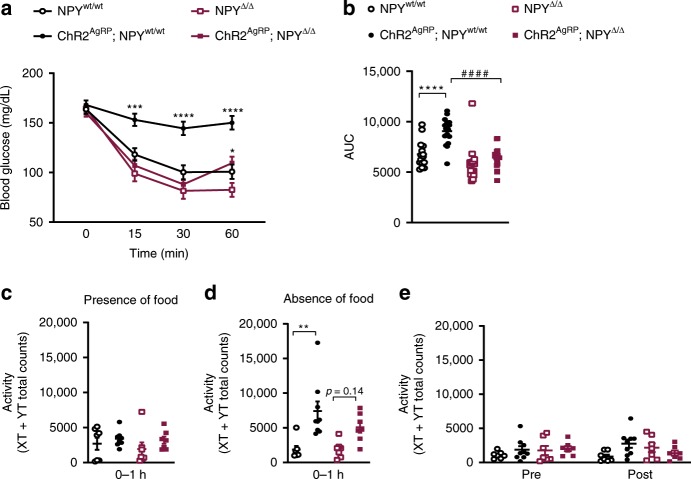
NPY is necessary for the acute insulin resistance upon optogenetic activation of AgRP neurons. **a, b** Blood glucose levels in NPY-deficient and control mice during insulin tolerance tests and area under the curve (AUC); *n* = 15 mice for NPY^wt/wt^, *n* = 17 mice for ChR2^AgRP^; NPY^wt/wt^ and ChR2^AgRP^; NPY^Δ/Δ^ and *n* = 18 mice for NPY^Δ/Δ^). **c**, **d** Locomotor activity during optogenetic AgRP neuron stimulation in the presence and in the absence of food is largely independent of NPY expression (*n* = 7 mice for NPY^wt/wt^, NPY^Δ/Δ^ and ChR2^AgRP^; NPY^Δ/Δ^ and *n* = 9mice for ChR2^AgRP^; NPY^wt/wt^). **e** Locomotor activity one hour prior light illumination of the ARC (pre) and one hour after laser was turned off (post) is similar in all genotypes (*n* = 7 mice for NPY^wt/wt^, NPY^Δ/Δ^ and ChR2^AgRP^; NPY^Δ/Δ^, and *n* = 9 mice for ChR2^AgRP^; NPY^wt/wt^). Data are shown as mean ± s.e.m. Statistical analysis is represented by *p ≤ 0.05, **p ≤ 0.01,***p ≤ 0.001, ****p ≤ 0.0001, and ^####^*p* ≤ 0.0001 as determined by two-way ANOVA followed by Tukey post hoc test, except in **b** and **d** in which a one-way ANOVA followed by Tukey post hoc test was used. # represents comparisons between NPY-expressing and NPY-deficient animals. Source data are provided as a Source Data file.

Since stimulation of AgRP neurons not only increases feeding but also induces systemic insulin resistance^7^, we next investigated the contribution of NPY signaling to the insulin resistance-inducing effect of AgRP neuron activation in the different groups of mice. This analysis revealed that optogenetic stimulation of AgRP neurons in ChR2^AgRP^; NPY^wt/wt^ mice resulted in the induction of clear insulin resistance during an insulin tolerance test (ITT; Fig. 4a, b, Supplementary Fig. 3a, b). In contrast, optogenetic stimulation of AgRP neurons in ChR2^AgRP^; NPY^Δ/Δ^ mice, which express ChR2 in AgRP neurons, but lack NPY, failed to affect the initial drop in blood glucose concentrations during the ITT (Fig. 4a, b, Supplementary Fig. 3a, b). However, in these mice, blood glucose concentrations during the ITT increased slightly at the 60 min time point as compared with NPY^Δ/Δ^ mice (Fig. 4a, b, Supplementary Fig. 3a, b). Of note, upon light illumination of the ARC, NPY^Δ/Δ^ mice as compared with control NPY^wt/wt^ mice presented a slight, yet non-significant increase in insulin sensitivity (Fig. 4a, b, Supplementary Fig. 3a, b). Assessment of insulin sensitivity in the four different groups of mice in the absence of blue light illumination revealed a minor improvement of insulin sensitivity at 60 min in NPY^Δ/Δ^ mice compared to the other groups of mice (Supplementary Fig. 3c–f). Nevertheless, these data collectively demonstrate that NPY deficiency largely abrogated the profound insulin resistance induced by optogenetic stimulation of AgRP neurons.

Since activation of AgRP neurons in absence of food increases locomotor activity in a rapid manner^6,7^, we next addressed whether NPY mediates this effect. Of interest, while optogenetic stimulation of AgRP neurons in both ChR2^AgRP^; NPY^wt/wt^ and ChR2^AgRP^; NPY^Δ/Δ^ mice failed to affect locomotor activity in the presence of food, the same stimulation increased locomotor activity in the absence of food in ChR2^AgRP^; NPY^wt/wt^, and there was a similar response in ChR2^AgRP^; NPY^Δ/Δ^ mice (Fig. 4c, d). As with the food intake, locomotor activity pre and post light illumination did not differ between all four groups of animals (Fig. 4e). Thus, increased locomotor activity upon AgRP neuron stimulation in the absence of food is largely independent of NPY signaling.

### Selective re-expression of NPY in AgRP neurons

**Fig. 5:**
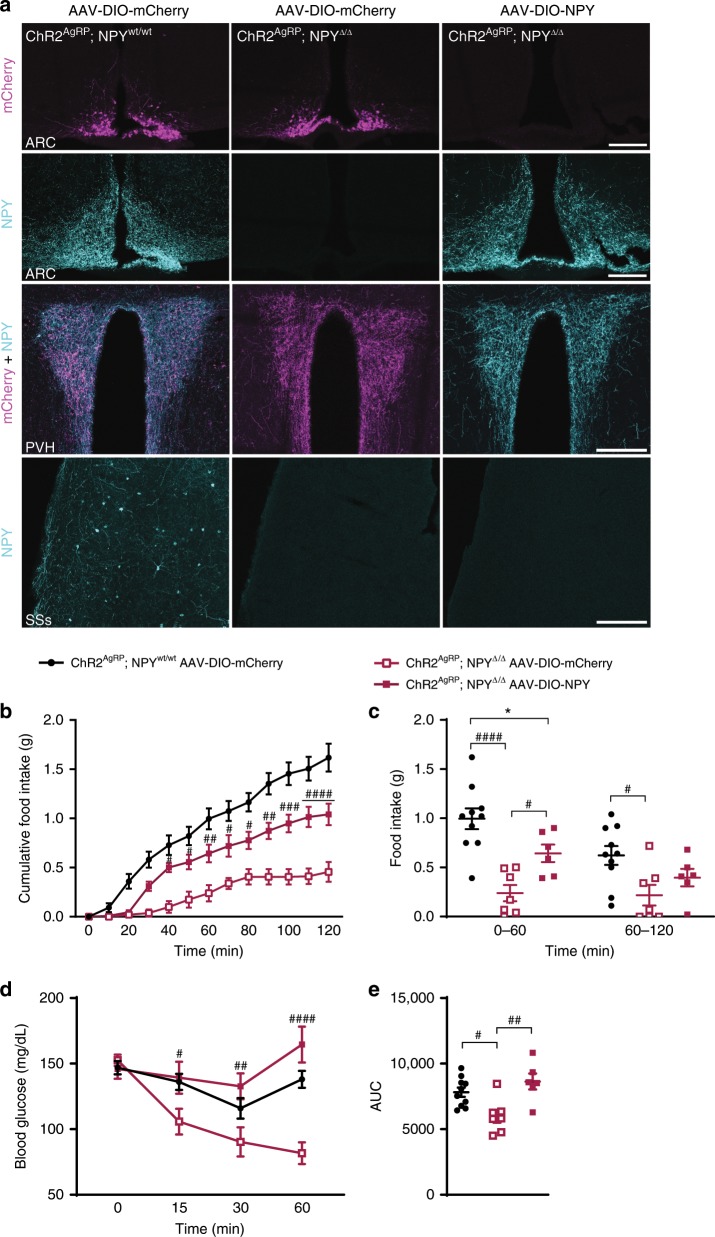
Virally transduced re-expression of NPY selectively in AgRP neurons of NPY knockout mice restores the feeding response and insulin resistance upon optogenetic activation of AgRP neurons. **a** Microphotographs show the successful expression of bilaterally delivered control virus (AAV-DIO-mCherry) in ChR2^AgRP^; NPY^wt/wt^ and ChR2^AgRP^; NPY^Δ/Δ^ mice, and NPY virus (AAV-DIO-NPY) in ChR2^AgRP^; NPY^Δ/Δ^ mice in the ARC. Note the presence of NPY containing fibers in the PVH of ChR2^AgRP^; NPY^Δ/Δ^ mice re-expressing NPY, which is absent in the corresponding control. In contrast, the supplementary somatosensory area of the cortex (SSs), which receives no projections from the ARC, displays NPY labeling only in ChR2^AgRP^; NPY^wt/wt^ mice. Scale bar = 200 µm. **b**, **c** Cumulative and total food intake upon AgRP neuronal stimulation in virally transduced NPY-deficient and wildtype control mice (mCherry) and in NPY-deficient mice re-expressing NPY in the ARC. **d**, **e** Blood glucose levels upon AgRP neuronal activation during insulin tolerance tests and corresponding area under the curve (AUC) in virally transduced NPY-deficient and wildtype control mice (mCherry) and in NPY-deficient mice re-expressing NPY in the ARC. *n* = 10 and *n* = 7 mice for ChR2^AgRP^; NPY^wt/wt^ and ChR2^AgRP^; NPY^Δ/Δ^ injected with mCherry virus and *n* = 6 mice for ChR2^AgRP^; NPY^Δ/Δ^ injected with NPY virus. Data are shown as mean ± s.e.m. Statistical analysis is represented by *p ≤ 0.05, ^#^*p* ≤ 0.05, ^##^*p* ≤ 0.01, ^###^*p* ≤ 0.001, and ^####^*p* ≤ 0.0001 as determined by two-way ANOVA (panels **b**–**d**) followed by Tukey post hoc test, or one-way ANOVA (panel **e**) followed by Tukey post hoc test. In panels **b** and **d**, # represents comparisons between NPY re-expressing mice and NPY-deficient mice injected with control virus. Source data are provided as a Source Data file.

Since NPY is expressed not only in AgRP neurons, but in a wider group of cells in both, the brain and peripheral tissues, we next aimed at investigating whether the observed responses were the specific results of NPY expression in AgRP neurons. To this end, we stereotactically delivered an AAV allowing for Cre-dependent expression of NPY in the ARC of ChR2^AgRP^; NPY^Δ/Δ^ mice. As control animals served ChR2^AgRP^; NPY^wt/wt^ and ChR2^AgRP^; NPY^Δ/Δ^ mice that were injected with an AAV allowing for Cre-dependent expression of mCherry. Immunohistochemical analysis revealed successful expression of NPY in the ARC of ChR2^AgRP^; NPY^Δ/Δ^ mice and mCherry expression in the ARC of control animals (Fig. 5a). Next, we compared the feeding stimulatory action of AgRP neurons upon optogenetic activation in the different groups of mice. While the acute effects on food intake were largely attenuated in control animals expressing mCherry in AgRP neurons, re-expression of NPY in ChR2^AgRP^; NPY^Δ/Δ^ mice partially restored the acute increase in food intake upon optogenetic activation of AgRP neurons (Fig. 5b, c, Supplementary Fig. 4a, b) confirming prior findings^11^. Strikingly, selective re-expression of NPY in AgRP neurons in ChR2^AgRP^; NPY^Δ/Δ^ mice completely restored the induction of insulin resistance upon optogenetic activation of AgRP neurons during an ITT (Fig. 5d, e, Supplementary Fig. 5a–f). As expected, stimulation of AgRP neurons in control ChR2^AgRP^; NPY^wt/wt^ animals expressing mCherry in the ARC resulted in the induction of insulin resistance during an ITT while ChR2^AgRP^; NPY^Δ/Δ^ mice expressing mCherry in AgRP neurons exhibited a profound protection from the induction of insulin resistance during the ITT (Fig. 5d, e, Supplementary Fig. 5a–f). Collectively, these experiments demonstrate that NPY expression in AgRP neurons is required for the acute food intake stimulatory and insulin resistance-inducing effects of optogenetic AgRP neuron activation.

### Chemogenetic activation of AgRP neurons in the absence of NPY

**Fig. 6:**
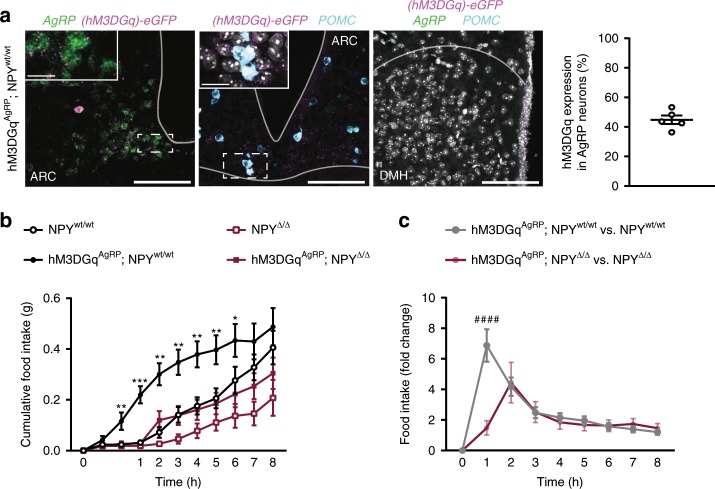
NPY-dependent signaling mediates the increase in feeding as well as the induction of insulin resistance upon chemogenetic stimulation of AgRP neurons. **a** Expression of *hM3DGq* occurs exclusively in AgRP neurons of the ARC. DAPI is depicted in gray in the DMH image (*n* = 5 mice). Scale bar = 100 µm; applies to all images; scale bar = 20 µm; applies to insets. **b** Food intake upon hM3DGq-induced stimulation of AgRP neurons in the presence and in the absence of NPY (*n* = 10 mice for NPY^wt/wt^, hM3DGq^AgRP^; NPY^wt/wt^ and NPY^Δ/Δ^, and *n* = 11 mice for hM3DGq^AgRP^; NPY^Δ/Δ^). **c** NPY is necessary for the acute food consumption upon AgRP neuron stimulation (*n* = 10 mice for NPY^wt/wt^, hM3DGq^AgRP^; NPY^wt/wt^ and NPY^Δ/Δ^, and *n* = 11 mice for hM3DGq^AgRP^; NPY^Δ/Δ^). All animals were injected with CNO. Data are shown as mean ± s.e.m. Statistical analysis is represented by *p ≤ 0.05, **p ≤ 0.01, ***p ≤ 0.001, and ^####^*p* ≤ 0.0001 as determined by two-way ANOVA followed by Tukey post hoc test, except for panel **c** where a Sidak post hoc test was performed. # represents comparisons between NPY-expressing and NPY-deficient animals. Source data are provided as a Source Data file.

To further confirm the role of NPY-dependent and NPY-independent signaling of AgRP neurons through a complementary approach, we investigated the consequences of chemogenetically activating AgRP neurons in the presence and absence of NPY expression. To this end, we expressed the stimulatory chemogenetic receptor hM3DGq from the ROSA26 locus (ROSA26hM3DGq) in a Cre-dependent manner in AgRP neurons of mice expressing or lacking NPY. Intercrosses yielded littermate mice, which were either controls (ROSA26hM3DGq^fl/fl^; AgRPCre^wt/wt^; NPY^wt/wt^, i.e. NPY^wt/wt^), which expressed hM3DGq in AgRP neurons in the presence of NPY (ROSA26hM3DGq^fl/fl^; AgRPCre^Cre/wt^; NPY^wt/wt^, i.e. hM3DGq^AgRP^; NPY^wt/wt^), which lacked NPY expression in the absence of hM3DGq expression in AgRP neurons (ROSA26hM3DGq^fl/fl^; AgRPCre^wt/wt^; NPY^Δ/Δ^, i.e. NPY^Δ/Δ^) or those expressing hM3DGq in AgRP neurons in the absence of NPY expression (ROSA26hM3DGq^fl/fl^; AgRPCre^Cre/wt^; NPY^Δ/Δ^, i.e. hM3DGq^AgRP^; NPY^Δ/Δ^) (Fig. 1a). To assess the specificity of AgRP-neuron-restricted hM3DGq-expression in hM3DGq^AgRP^-mice, we performed an analysis of mRNA expression of *eGFP*, which is co-expressed with hM3DGq in a Cre-dependent manner in these mice as well as analyses of *AgRP* and *POMC* mRNA expression. This analysis revealed that 45% of AgRP-expressing neurons in the ARC expressed hM3DGq, while hM3DGq expression was not detectable in POMC-expressing neurons in the ARC, nor in neurons of the DMH (Fig. 6a). Similar to what we observed in mice expressing ChR2 in AgRP neurons, also the expression of hM3Dq in the presence or absence of NPY did not cause any differences in *AgRP* mRNA expression levels between animals of all four genotypes as assessed by qPCR, and confirmed the successful inactivation of NPY in NPY^Δ/Δ^ mice (Supplementary Fig. 6a, b).

We first investigated the ability of an intraperitoneal injection of Clozapine-*N*-oxide (CNO; 0.3 mg/kg BW) to induce light-cycle feeding in these animals. These experiments revealed that hM3DGq-induced activation of AgRP neurons rapidly increased light-cycle feeding in hM3DGq^AgRP^; NPY^wt/wt^ mice, resulting in a six-fold increase when compared with NPY^wt/wt^ littermate controls during the first 60 min after CNO injection (Fig. 6b, c). In contrast, hM3DGq-induced activation of AgRP neurons in hM3DGq^AgRP^; NPY^Δ/Δ^ mice, which lack NPY, failed to significantly induce food intake as compared to NPY^Δ/Δ^ control mice during this period of time (Fig. 6b, c). Additionally, CNO injection increased food intake to the same relative magnitude from 2 h after CNO injection onwards in hM3Dq^AgRP^; NPY^wt/wt^ as compared with NPY^wt/wt^ as well as between hM3Dq^AgRP^; NPY^Δ/Δ^ compared to NPY^Δ/Δ^ mice (Fig. 6b, c). In contrast, control experiments revealed that the immediate food intake upon vehicle injection was similar in all groups of mice (Supplementary Fig. 6c). Thus, consistent with the lower proportion of AgRP neurons expressing hM3DGq in hM3DGq^AgRP^ mice (Fig. 6a), compared to the proportion of AgRP neurons expressing ChR2 in ChR2^AgRP^ mice (Fig. 1b), the absolute magnitude of food intake stimulation was lower in this model. Nevertheless, these findings further substantiate the exclusive dependence of the immediate feeding response to AgRP neurons activation on NPY expression, while the prolonged increase of feeding occurs independently of NPY-dependent signaling.

**Fig. 7:**
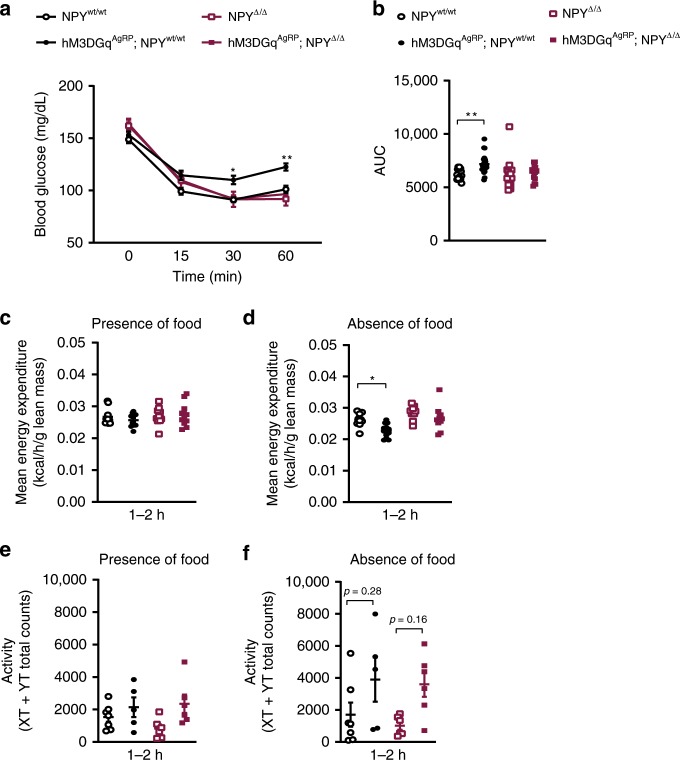
NPY is necessary for the acute insulin resistance upon chemogenetic activation of AgRP neurons. **a**, **b** Insulin tolerance test in NPY-expressing and NPY-deficient mice and corresponding area under the curve (AUC); *n* = 17 mice for NPY^wt/wt^, *n* = 23 mice for hM3DGq^AgRP^; NPY^wt/wt^, *n* = 14 mice for NPY^Δ/Δ^ and *n* = 15 mice for hM3DGq^AgRP^; NPY^Δ/Δ^). **c** Energy expenditure in the presence of food (1–2 h post injection) is similar in all groups of mice (*n* = 10 mice for NPY^wt/wt^, hM3DGq^AgRP^; NPY^wt/wt^ and NPY^Δ/Δ^, and *n* = 11 mice for hM3DGq^AgRP^; NPY^Δ/Δ^). **d** Energy expenditure in the absence of food (1–2 h post injection) is dependent on NPY expression (*n* = 10 mice for NPY^wt/wt^, *n* = 12 mice for hM3DGq^AgRP^; NPY^wt/wt^, *n* = 9 mice for NPY^Δ/Δ^ and *n* = 10 mice for hM3DGq^AgRP^; NPY^Δ/Δ^). **e** Locomotor activity in the presence of food (1–2 h post injection) is similar in all groups of mice (*n* = 7 for NPY^wt/wt^, *n* = 5 for hM3DGq^AgRP^; NPY^wt/wt^ and *n* = 6 for NPY^Δ/Δ^ and hM3DGq^AgRP^; NPY^Δ/Δ^). **f** Locomotor activity in the absence of food (1–2 h post injection) is largely independent of NPY (*n* = 7 for NPY^wt/wt^, *n* = 5 for hM3DGq^AgRP^; NPY^wt/wt^ and *n* = 6 for NPY^Δ/Δ^ and hM3DGq^AgRP^; NPY^Δ/Δ^). All animals were injected with CNO. Data are shown as mean ± s.e.m. Statistical analysis is represented by *p ≤ 0.05 and **p ≤ 0.01 as determined by one-way ANOVA followed by Tukey post hoc test, except for panel **a**, where a two-way ANOVA followed by Tukey post hoc test was performed. Source data are provided as a Source Data file.

Next, we investigated the ability of chemogenetic AgRP neuron stimulation to induce systemic insulin resistance in the presence or absence of NPY expression. Consistent with our optogenetic experiments, chemogenetic stimulation of AgRP neurons in hM3DGq^AgRP^; NPY^wt/wt^ mice, as compared with control NPY^wt/wt^ mice, induced systemic insulin resistance (Fig. 7a, b, Supplementary Fig. 7a, b). The induced insulin resistance upon chemogenetic stimulation was milder than that induced by optogenetic stimulation of AgRP neurons, which is again consistent with the lower proportion of AgRP neurons expressing hM3DGq in this mouse model (Figs. 6a and  1b). However, the insulin resistance inducing effect of chemogenetic AgRP neuron stimulation was completely abrogated in hM3Dq^AgRP^ mice lacking NPY expression (hM3DGq^AgRP^; NPY^Δ/Δ^), compared to both NPY^wt/wt^ and NPY^Δ/Δ^ mice (Fig. 7a, b, Supplementary Fig. 7a, b). Of note, mice of all four genotypes exhibited a comparable degree of insulin resistance upon vehicle injection (Supplementary Fig. 7c–f). Thus, similar to what we observed in our optogenetic studies, the insulin resistance inducing effect of AgRP neuron activation largely depends on NPY signaling.

Finally, we compared the effect of chemogenetically activating AgRP neurons in the presence or absence of NPY on energy expenditure. Of interest, while energy expenditure was similar in all groups of mice when food was present upon CNO injection (Fig. 7c), hM3DGq-induced stimulation of AgRP neurons induced a decrease of energy expenditure in the absence of food in mice expressing NPY (hM3DGq^AgRP^; NPY^wt/wt^), but not in mice lacking NPY (hM3DGq^AgRP^; NPY^Δ/Δ^) (Fig. 7d). Moreover, assessment of locomotor activity in the presence of food revealed no difference between the different genotypes upon CNO injection (Fig. 7e). In contrast, there was a slight increase in locomotor activity in both hM3DGq^AgRP^; NPY^wt/wt^, as well as in hM3DGq^AgRP^; NPY^Δ/Δ^-mice upon CNO administration (Fig. 7f).

## Discussion

AgRP-expressing neurons represent an integral component of a homeostatic neurocircuitry adapting a wide range of physiological responses in accordance with the energy state of the organism, including the regulation of feeding^4^, insulin sensitivity^9,7^, immune responses^17^, bone mineral density^18^, and sensation of pain^19^. Ablation of these neurons in adult mice induces starvation due to disinhibition of CGRP-expressing neurons in the lateral parabrachial nucleus^20,21^, while their chemogenetic or optogenetic stimulation evokes voracious feeding. They are activated upon fasting, inhibited upon refeeding as well as transiently and rapidly inhibited during the sensory perception of food^2^. Here, sensory food perception-dependent regulation of AgRP neurons controls BAT thermogenesis^22^. Interestingly, the ability of sensory food perception to simultaneously activate POMC neurons has been linked to prime liver ER-homeostasis for the postprandial state, in line with the general concept, that melanocortin neurons adapt multiple physiological processes in accordance to anticipated or actual changes in the energy state of the organism^23^.

Neurocircuitry mapping experiments have demonstrated that AgRP neurons exert their metabolism regulatory function via a wide range of projections exerting partly distinct and overlapping regulatory functions to mediate their pleiotropic effects^24,7^. In addition to expressing their characteristic neuropeptide AgRP, which acts as an inverse agonist on the melanocortin 4 receptor (MC4R), upon activation they also release NPY and GABA. The importance of GABA release from these cells in the long-term control of feeding and glucose homeostasis has been demonstrated through both constitutive and inducible targeted ablation of the vesicular GABA transporter (VGAT) from these neurons^14,25^. Moreover, injection of a GABA receptor antagonist as well as an NPY1 receptor antagonist in the PVH prior to optogenetic stimulation of AgRP terminals attenuates the feeding stimulatory effect of activating this projection site^15^. Interestingly, a previous study reported that chemogenetic stimulation of AgRP neurons in either NPY-deficient mice or in mice lacking VGAT selectively in AgRP neurons failed to attenuate the rapid feeding response, while the combined deficiency for both VGAT and NPY abrogated the immediate feeding response^10^. In contrast, acute increases in food intake upon optogenetic stimulation of AgRP neurons were dramatically reduced in NPY-deficient mice as revealed in a recent study^11^. Here, we found that deficiency of NPY is sufficient to abrogate virtually completely the immediate feeding and glucose homeostasis regulatory function of AgRP neurons in two complementary models, namely chemogenetic and optogenetic stimulation. A potential limitation of all of these studies is the use of conventional NPY-deficient mice. These animals lack NPY not only in brain sites with metabolism regulatory functions, but also in peripheral organs implicated in the control of glucose metabolism^26,27^. However, in the present study we have selectively activated AgRP neurons through complementary chemogenetic and optogenetic approaches and detected changes in AgRP-driven food intake and insulin resistance, which we did not detect in the respective NPY proficient and deficient control groups. Further, we demonstrate that re-expression of NPY selectively in AgRP neurons restores both, increase in feeding and the induction of insulin resistance, upon optogenetic activation of AgRP neurons. Thus, the observed phenotypes likely result from AgRP neuron-dependent NPY release. Also, we find clear differences in the magnitude by which optogenetic versus chemogenetic activation of AgRP neurons promotes feeding and induces insulin resistance. However, these differences are well explained through the different efficiencies with which hM3Dq- and ChR2-channels are expressed in the two different mouse models dependent on Cre-mediated recombination, likely explained by the different constructs promoting expression of hM3Dq and ChR2 from the modified ROSA 26 locus employing different enhancers^12,7^. In contrast to our transgenic approaches, the previous study by Krashes et al. had employed virus-based, and presumably, very high level expression of hM3DGq in AgRP neurons, while both our optogenetic and chemogenetic approaches utilized lower level, homogenous expression of ChR2 and hM3DGq in AgRP neurons of transgenic mice^10^. Whether this technical difference or other factors contribute to the observed phenotypical differences will have to be examined in future studies. Furthermore, consistent with a role for NPY in the rapid response to refeeding after a fasting period when AgRP neuron activity is high, we find an attenuated refeeding response in the first hour in mice lacking NPY similar to that observed in mice lacking NPY-1 and NPY-5 receptors^28^. In contrast, the rate of feeding induction in a prolonged manner appears to be independent of NPY-evoked signaling. Thus, our data clearly reveal an important role for NPY release from AgRP neurons in acutely promoting feeding, while the later response likely occurs as a consequence of AgRP released from these neurons. This conclusion is consistent with both the effect of blocking MC4R signaling upon chemogenetic activation of AgRP neurons and the prolonged feeding-stimulatory effect of a single intracerebroventricular (icv) injection of AgRP^10,29^.

Further, we demonstrate that the acute insulin resistance-inducing effect of both chemogenetic and optogenetic AgRP neuron activation strongly depends on NPY signaling. We had previously demonstrated that the insulin resistance-inducing effect of AgRP neuron activation occurs in part through an acute suppression of sympathetic nerve activation (SNA) of brown adipose tissue (BAT) to induce myostatin expression in BAT^7^. Consistent with the present data that the ability of AgRP neurons to induce insulin resistance depends on NPY expression, icv injection of NPY has been demonstrated to rapidly and profoundly reduce BAT SNA^30,31^ as well as to improve systemic insulin sensitivity^32^. Thus, the present study clearly sets the ground to further define the exact molecular nature of NPY receptor expressing neurons acting downstream of AgRP neurons to mediate the impairment of systemic insulin sensitivity. In addition, the NPY-dependent regulation of systemic insulin sensitivity, presumably via regulation of BAT SNA, is consistent with the reduction of energy expenditure in mice in the absence of food, when AgRP neurons are activated chemogenetically in the presence but not in the absence of NPY.

Finally, our studies reveal that the acute locomotor-inducing activity of AgRP neuron activation occurs largely independent of NPY. This is interesting in light of the general notion, that AgRP-dependent activation of locomotion represents a foraging component of food intake-related behavior. Given that the acute food intake stimulatory effect of AgRP neuron activation depends on NPY, while the induction of locomotor activity does largely not, points to the possibility, that both behaviors are differentially encoded and thus not necessarily functionally linked. Collectively, our findings identify the distinct role for NPY-dependent AgRP neuronal function in the acute regulation of feeding and insulin sensitivity, while prolonged feeding responses and locomotor regulatory functions of AgRP neurons appear to occur in an NPY-independent manner.

## Methods

### Animal care

All experiments have complied with all relevant ethical regulations for animal testing and research. All animal procedures were conducted according to the protocols approved by the local government authorities (Bezirksregierung Köln). Mice were group housed (3–5 animals per cage) in a controlled environment with regards to humidity and temperature (22–24 ^o^C) on a 12 h light/12 h dark cycle. Mice destined for the fasting–refeeding experiment, indirect calorimetry, and for optogenetics (post-surgery) were kept single-housed. Mice had ad libitum access to water, and to a standard rodent chow (ssniff^®^, V1554), unless food was withdrawn for a specific experiment. All experiments were performed in adult mice, age 11–19 weeks (chemogenetics) or 10–16 weeks (optogenetics). For chemogenetic experiments, only male mice were used. For optogenetic experiments, both genders were employed, always considering proper balance with respect to gender for all genotypes.

### Mouse lines

All mouse lines used have been previously described; AgRP-IRES-cre^14^, hM3D_Gq_^fl/fl^ (ROSA26CAGSTOPloxSTOPloxhM3DGq;^7^, ChR2^fl/fl^ (ROSA26CAGSTOPloxSTOPloxChR2[H134R]-EYFP-WPRE; Ai32;;^12^ NPY^∆/∆^^13^. The primers used for AgRP were: AgRP1 (GGGCCCTAAGTTGAGTTTTCCT), AgRP2 (GATTACCCAACCTGGGCAGAAC), and AgRP3 (GGGTCGCTACAGACGTTGTTTG). The primers used for hM3DGq were: CAGS_Fow (AAAGTCGCTCTGAGTTGTTATC), CAGS_RevWT (GATATGAAGTACTGGGCTCTT), and CAGS_RevWT (TGTCGCAAATTAACTGTGAATC). The primers used for ChR2 were: Ai27-1-F1_5′ (AAAGTCGCTCTGAGTTGTTATC), Ai27-1-R1_5′ (GATATGAAGTACTGGGCTCTT), and Ai27-1-R3 (TTACTATGGGAACATACGTCAT). The primers used for NPY^∆/∆^ were: 12265_Mutant reverse (GGCCTCTTCGCTATTACGC), 23324_Common (ACGGTCGGGGATAGAGAGAG), and 23325_WT Reverse (CAAGTTCACTGGCGTCTGG).

### Generation of experimental mice

In order to chemogenetically activate AgRP neurons on an NPY^wt/wt^ or NPY^∆/∆^ background, AgRP-IRES-Cre mice were bred with hM3DGq^fl/fl^ mice, and the offspring crossed with NPY^∆/∆^ mice. Mice from these breedings were further crossed with hM3DGq^fl/fl^ mice in order to generate homozygosity for the hM3DGq allele. To generate the four experimental groups, hM3DGq^fl/fl^; AgRPCre^Cre/wt^; NPY^∆/wt^ mice were bred with hM3DGq^fl/fl^; AgRP-Cre^wt/wt^; NPY^∆/wt^. Mice for the optogenetic experiments were generated in a similar way, by crossing AgRPCre^Cre/wt^ mice with ChR2^fl/fl^ mice and subsequently with the NPY^∆/∆^ line. The resulting four experimental groups were all heterozygous for ChR2. All mouse lines were maintained on a C57/Bl6 background with the exception of NPY-deficient mice, which were on a 129sv background.

### Surgery

Mice were anesthetized with isoflurane and received an i.p. bolus of Buprenorphin (0.1 mg/kg BW), and were put into a stereotaxic frame (David Kopf Instruments). A local anesthetic agent (Lidocaine) was applied to the skin, the skull surface was exposed through a skin incision, and a small drill hole was made. A flat tip fiber-optic cannula (6 mm long, 200 µm in core diameter, numerical AP 0.48; Doric lenses Inc.) was inserted (coordinates from Bregma: −1.6 AP, 0.2 ML, and −5.2 DV) and secured to the skull with dental acrylic (diluted Super Bond C&B). For re-expression of NPY in the ARC, bilateral injections of an AAV8-EF1a-DIO-NPY virus (kindly provided by Dr. Z. Knight^11^) or control virus AAV8-hSyn-DIO-mCherry (Addgene, cat# 50459-AAV8, lot# v43120) were made immediately before the insertion of the fiber-optic cannula (300 nL; coordinates from Bregma: −1.6 AP, ±0.3 ML and −6 to −5.8 DV). Before waking up, mice were given a subcutaneous injection of Meloxicam (5 mg/kg) for post-operative pain relief. Mice were carefully monitored after the surgery, and body weight was measured daily for 1 week. All virus injections and fiber-optic cannula placements were histologically verified after the experiments. Animals in which the virus expression was very low/absent or in which the fiber-optic cannula was not correctly placed were excluded from the study.

### In vivo photostimulation

Before the optogenetic experiments, mice were allowed to recover for at least one week post-surgery. Mice with bilateral virus injections were allowed to recover for 3 weeks. They were then put into experimental cages for habituation to food hoppers and water dispensers. All mice were handled on a daily basis to reduce stress during the subsequent experimental procedures. After approximately one week, they were connected to a fiber-optic patch cord (core diameter 200 µm, numerical AP 0.48; Doric lenses) connected to a rotary joint (Doric lenses), and allowed to adapt to this for another period of 3–4 days. On the experimental day, at the beginning of the light phase, the attached fiber-optic patch cord was replaced by a new one. The protocol for photostimulation followed^5^, in which laser light was applied repeatedly over 1 s followed by a 3 s break. The photostimulation frequency was 20 Hz with 10 ms light pulses. A laser power of 20 mW was used, rendering an irradiance of ~3–7 mW/mm^2^ in the targeted region, as calculated with the online tool at https://web.stanford.edu/group/dlab/cgi-bin/graph/chart.php, hence above the threshold for activation of ChR2 (~1 mW/mm^2^^33^). As confirmed with RNAscope, this stimulation gave rise to consistent and bilateral activation of AgRP neurons (see Fig. 1b, c). All optogenetic experiments were initiated 3 h into the light phase. At the time of the experiments, all mice were stable in body weight.

### Fluorescent in situ hybridization

Mice implanted with fiber-optic cannulas were photostimulated in the absence of food for 60 min, then deeply anesthetized and transcardially perfused with 0.9% saline, followed by ice-cold 4% phosphate-buffered PFA. The heads were placed into 4% phosphate-buffered PFA for 4 h. Cannulas and brains were removed and transferred into 4% phosphate-buffered PFA for post fixation for 18 h at room temperature. Subsequently the brains were transferred to sterile PBS containing 25% sucrose and kept in this solution at 4 ^o^C for 24 h. Brains were cut at 14 µm on a freezing microtome, collected in sterile bins containing cryoprotectant and stored at −20 ^o^C until further use.

On the day before the assay, every 12th section throughout the ARC was mounted onto SuperFrost Plus Gold slides (ThermoFisher), dried at room temperature and then incubated at 60 ^o^C o/n. For the simultaneous detection of *Fos* and *AgRP* mRNA and of simultaneous detection of *AgRP*, *eGFP*, and *POMC* we utilized fluorescent RNAscope^®^ (ACD; Advanced Cell Diagnostics Inc., Hayward, CA). All reagents were purchased from ACD unless otherwise stated. The *Fos* probe-targeted region 407–1427, access # NM_010234.2. The *AgRP* probe-targeted region 11–764, access # NM_001271806.1. The *eGFP* probe-targeted region 628–1352, access # NM U55763.1. Of note, eGFP probe was used to detect eYFP because it recognizes 100% of the eYFP. The POMC probe-targeted region 19–995, access code # NM_008895.3. Negative and positive control probes recognizing dihydrodipicolinate reductase, DapB (a bacterial transcript) and cyclophilin and PolR2A, respectively, were processed in parallel with the target probes to ensure tissue RNA integrity and optimal assay performance. All incubation steps were performed at 40 ^o^C using a humidified chamber and a HybEz oven. The pre-treatment protocol has been described previously^23^ and the detection protocol followed the manufacturer’s instructions for the RNAscope Multiplex Fluorescent v2 kit. For detection of the probes directed towards *Fos* and *AgRP* mRNA, Cy3, and Opal520 tyramides were used (PerkinElmer), dissolved in DMSO according to the manufacturer’s instructions, and diluted 1:3000 and 1:2000 for working solutions, respectively. For simultaneous detection of *AgRP*, *eGFP*, and *POMC*, probes and respective tyramides working solutions were diluted as follows: *AgRP* (1:100)-Opal520 (1:1000), *eGFP* (1:50)-Cy3 (1:750), and *POMC* (1:100)-Cy5 (1:3000).

### Immunohistochemistry

Brains from mice with bilateral virus injections were harvested and post-fixed as described above, and cut at 30 µm on a freezing microtome. For the detection of NPY, a monoclonal rabbit anti-NPY antibody (1:1000; cat# 11976, Cell Signaling Technology) was used, visualized with an Alexa 647 donkey anti-rabbit secondary antibody (1:500, cat# A31573, Invitrogen). The endogenous fluorescence from mCherry was evident without any immunohistochemical detection.

### Imaging and quantification

Images for the quantification of RNAscope data were captured using a confocal Leica TCS SP-8-X microscope, equipped with a ×40/1.30 oil objective. Tile scans and Z-stacks (optical section of 1.0 μm) of 4–5 sections containing the ARC per animal were captured unilaterally from rostral to caudal. Laser intensities for the different channels were kept constant throughout the imaging process. Maximum intensity projections were made in FIJI (NIH) and the DAPI signal was adjusted with respect to contrast and brightness. The probe channels were not modified. The channels were fused and imported into the Halo software (Indica Labs), which utilizes the DAPI stain for automated cellular recognition. Based on each defined cell, and the detected probe signal within each cell, the software calculates the cell intensity, which is an integrated number of the intensity of the probe signal and the area of each cell covered by the probe. For simultaneous detection of *AgRP*, *eGFP*, and *POMC*, the counting was performed manually, using DAPI as a reference for co-localization of *AgRP/POMC* signal with *eGFP* signal. Between 3 and 6 hemisections were quantified per animal. For microphotographs in Fig. 1c, one section from each genotype was imaged with the same confocal and objective; at a resolution of 1024 × 1024 and a scan speed of 200 ms. For Fig 5, the PVH and the ARC from one animal of each experimental group were imaged with a ×20/0.75 imm objective, 400 ms scan speed. Images were cropped and edited in Adobe Photoshop CC with regards to brightness and contrast. The settings were applied equally across all images.

### qPCR

Mice were habituated to handling for one week prior to the experiment. On the day before the experiment, mice received a fresh cage. In the morning of the experimental day, food spills were removed from the cages. Approximately 3 h into the light-phase, mice were given an i.p. injection of CNO (Sigma Aldrich) and food was removed. After 60 min, they were decapitated and brains were quickly removed. A brain slice containing the hypothalamus was chilled on dry-ice and the ventral part of the hypothalamus was cut out and snap-frozen in liquid nitrogen. Total RNA was isolated using the mirVana Isolation Kit (Ambion), according to the manufacturer’s instructions. The RNA was reverse transcribed with the High-Capacity cDNA Reverse Transcription Kit (Applied Biosystems) using random hexamer primers. qPCR was perfomed using the Takyon LowRox MasterMix dTTP Blue (Eurogentec) and the QuantStudio 7 Flex Real-Time PCR System (Applied Biosystems). Hypoxanthine guanine phosphoribosyl transferase (Hprt) was used as house-keeping gene to normalize within each sample. The TaqMan probes employed were AgRP (Mm00475829_g1) and NPY (Mm00445771_m1) (Applied Biosystems). Data was analyzed using the ΔCt method.

### Electrophysiology

Mice were deeply anesthetized and decapitated, and brains were quickly removed into ice-cold cutting solution consisting of (in mM): 92 choline chloride, 30 NaHCO_3,_ 25 glucose, 20 HEPES, 10 MgSO_4_, 2.5 KCl, 1.25 NaH_2_PO_4_, 5 sodium ascorbate, 3 sodium pyruvate, 2 thiourea, 0.5 CaCl_2_ oxygenated with 95% O_2_/5% CO_2_, measured osmolarity 310–320 mOsm/L. 300 μm-thick coronal sections were cut with a Campden vibratome (Model 7000smz-2) and incubated in oxygenated cutting solution at 34 °C for 10 min. Slices were transferred to oxygenated aCSF consisting of (in mM; 126 NaCl, 21.4 NaHCO_3_, 2.5 KCl, 1.2 NaH_2_PO_4_, 1.2 MgCl_2_, 2.4 CaCl_2_, 10 glucose) at 34 °C for 30 min, and stored in the same solution at room temperature (20–24 °C) for at least 60 min prior to recording. A single slice was placed in the recording chamber where it was continuously superfused at a rate of 3–4 mL/min with oxygenated aCSF. Neurons were visualized with an upright microscope (SliceScope, Scientifica) equipped with infrared-differential interference contrast and fluorescence optics. Borosilicate glass microelectrodes (5–7 MΩ) were filled with a Cs^+^-based internal solution consisting of (in mM): 135 CsMeSO_3_, 10 HEPES, 1 EGTA, 4 MgCl_2_, 4 Na_2_-ATP, 0.4 Na_2_-GTP, 10 Na_2_-phosphocreatine (pH 7.3 adjusted with CsOH; 295 mOsm kg^−1^). To photostimulate ChR2-expressing AgRP terminals, an LED light source (473 nm) was focused onto the back aperture of the microscope objective, producing widefield exposure around recorded cells. Light-evoked IPSCs were recorded from PVH neurons in whole-cell voltage-clamp mode, with membrane potential clamped at *V*_h_ = 0 mV in presence of CNQX (10 µM) and D-AP5 (50 µM) to block glutamatergic synaptic transmission. Bicuculline (10 μM) was added to the aCSF to block GABAergic synaptic transmission. All recordings were made using a Multiclamp 700B amplifier, and data were filtered at 2 kHz and digitized at 10 kHz. The light-evoked IPSC detection protocol consisted of four blue light pulses (473 nm wavelength, 5 ms) administered 1 s apart during the first 4 s of an 8-s sweep. Evoked IPSCs with short latency (<10 ms) upon light stimulation and low jitter were considered light-driven. Light output was controlled by a programmable pulse stimulator, Master-8 (A.M.P.I.), and pClamp software (Axon Instruments). All recordings were analyzed offline using Clampfit.

### Food intake and locomotor activity

An automated food intake recording system, where each food hopper was connected to a sensor (TSE Systems), was used. Activity frames surrounding each cage allowed for simultaneous locomotor measurements (TSE Systems). Two days (optogenetics) or the day (chemogenetics) prior to the experiments, mice received fresh cages. On the experimental day, at the beginning of the light phase, food spills were removed from cages, and recordings were started 3 h later. Following 1 h of non-stimulated recordings (pre period), lasers were on for 2 h, followed by 1 h of non-stimulated recordings (post period) for optogenetics. Following CNO or vehicle (saline in the presence of DMSO) injection measurement started for chemogenetics.

### Locomotor activity in the absence of food

Two days (optogenetics) or one day (chemogenetics) before the experiments, mice were placed into fresh cages. At the beginning of the light phase on the experimental day, all food spills were carefully removed. Food hoppers were removed before light stimulation was initiated or CNO was administered, and activity frames were utilized for 60 min to determine locomotor activity (TSE Systems).

### Histology

Mice were decapitated, and heads were transferred into 4% phosphate-buffered formaldehyde (PFA). After at least 10 days, fiber-optic cannulas were removed, brains were dissected and transferred into 4% phosphate-buffered PFA containing 25% sucrose. After equilibration in this sucrose solution, brains were cut at 30 µm on a freezing microtome, stained with NeuroTrace™500/525 (1:250; ThermoFisher Scientific) and imaged for determination of fiber-optic cannula placements.

### Food intake and energy expenditure: Chemogenetics

For food intake and energy expenditure measurements, an open circuit indirect calorimetry system was used (PhenoMaster; TSE Systems). For the food intake recordings, mice previously single-housed and habituated to the PhenoMaster cages, food hoppers, and water dispensers were placed in the PhenoMaster chambers one day prior the experiment. On the experimental day, 3 h after beginning of the light cycle, mice were given an i.p. injection of CNO or vehicle and immediately returned back to their cages, where food intake and energy expenditure were automatically recorded. For measurements in the absence of food, mice were provided with a clean cage at the beginning of the light phase on the experimental day. Three hours later, food was removed, mice were injected i.p. with CNO, and immediately returned to their cages. Energy expenditure data was corrected for lean mass, as determined using an IVIS SpectrumCT scanner (Caliper LifeScience, USA)^34^.

### Insulin tolerance test

ITTs with chemogenetic or optogenetic stimulation of AgRP neurons were performed as previously described^7^. Mice received a clean cage one (chemogenetics) or two (optogenetics) days prior to the experiments. On the experimental day, at the beginning of the light phase, food spills were carefully removed from cages.

### Fasting and re-feeding

Male mice on NPY^wt/wt^ and NPY^∆/∆^ background were singly housed and provided with food hoppers for food intake measurements for, at least, 10 days prior to the experiments. At the beginning of the dark phase, mice received fresh cages without food. Following a 16 h fast, food hoppers filled with food were placed back into the cages and food intake was manually recorded at selected time points for 24 h.

### Statistics

Statistical analyses were performed using GraphPad Prism version 7 and significance was accepted when the *p* value was lower than 0.05. Two-way ANOVA was performed followed by a post hoc test (Tukey or Sidak, more details in the figure legends) when two variables were being compared. One-way ANOVA was performed when only one variable (genotype) was being compared.

### Reporting summary

Further information on research design is available in the Nature Research Reporting Summary linked to this article.

## Supplementary information

Supplementary Information

Reporting Summary

## Data Availability

The datasets generated during and/or analyzed during the current study are available from the corresponding author on reasonable request. The source data underlying Fig. 1b, d; 2a; 3a–d; 4a–e; 5b–e; 6a–c; and 7a–f and Supplementary Figs. 2a, b; 3a–f; 4 a, b; 5a–f; 6a–c; and 7a–f are provided as a Source Data file.

## Source data

Source Data

## References

[1] Ruud J, Steculorum SM, Bruning JC (2017). Neuronal control of peripheral insulin sensitivity and glucose metabolism. Nat. Commun..

[2] Chen Y, Lin YC, Kuo TW, Knight ZA (2015). Sensory detection of food rapidly modulates arcuate feeding circuits. Cell.

[3] Gropp E (2005). Agouti-related peptide-expressing neurons are mandatory for feeding. Nat. Neurosci..

[4] Luquet S, Perez FA, Hnasko TS, Palmiter RD (2005). NPY/AgRP neurons are essential for feeding in adult mice but can be ablated in neonates. Science.

[5] Aponte Y, Atasoy D, Sternson SM (2011). AGRP neurons are sufficient to orchestrate feeding behavior rapidly and without training. Nat. Neurosci..

[6] Krashes MJ (2011). Rapid, reversible activation of AgRP neurons drives feeding behavior in mice. J. Clin. Invest..

[7] Steculorum SM (2016). AgRP neurons control systemic insulin sensitivity via myostatin expression in brown adipose tissue. Cell.

[8] Bruning JC (2000). Role of brain insulin receptor in control of body weight and reproduction. Science.

[9] Konner AC (2007). Insulin action in AgRP-expressing neurons is required for suppression of hepatic glucose production. Cell Metab..

[10] Krashes MJ, Shah BP, Koda S, Lowell BB (2013). Rapid versus delayed stimulation of feeding by the endogenously released AgRP neuron mediators GABA, NPY, and AgRP. Cell Metab..

[11] Chen, Y. et al. Sustained NPY signaling enables AgRP neurons to drive feeding. *Elife* **8**, 10.7554/eLife.46348 (2019). 10.7554/eLife.46348 PMC6513552 31033437

[12] Madisen L (2012). A toolbox of Cre-dependent optogenetic transgenic mice for light-induced activation and silencing. Nat. Neurosci..

[13] Erickson JC, Clegg KE, Palmiter RD (1996). Sensitivity to leptin and susceptibility to seizures of mice lacking neuropeptide Y. Nature.

[14] Tong Q, Ye CP, Jones JE, Elmquist JK, Lowell BB (2008). Synaptic release of GABA by AgRP neurons is required for normal regulation of energy balance. Nat. Neurosci..

[15] Atasoy D, Betley JN, Su HH, Sternson SM (2012). Deconstruction of a neural circuit for hunger. Nature.

[16] Liu T (2012). Fasting activation of AgRP neurons requires NMDA receptors and involves spinogenesis and increased excitatory tone. Neuron.

[17] Matarese G (2013). Hunger-promoting hypothalamic neurons modulate effector and regulatory T-cell responses. Proc. Natl Acad. Sci. USA.

[18] Kim JG (2015). AgRP neurons regulate bone mass. Cell Rep..

[19] Alhadeff AL (2018). A neural circuit for the suppression of pain by a competing need state. Cell.

[20] Wu Q, Boyle MP, Palmiter RD (2009). Loss of GABAergic signaling by AgRP neurons to the parabrachial nucleus leads to starvation. Cell.

[21] Wu Q, Clark MS, Palmiter RD (2012). Deciphering a neuronal circuit that mediates appetite. Nature.

[22] Burke, L. K. et al. mTORC1 in AGRP neurons integrates exteroceptive and interoceptive food-related cues in the modulation of adaptive energy expenditure in mice. *Elife* **6**, 10.7554/eLife.22848 (2017). 10.7554/eLife.22848 PMC5441868 28532548

[23] Brandt C (2018). Food perception primes hepatic ER homeostasis via melanocortin-dependent control of mTOR activation. Cell.

[24] Betley JN, Cao ZF, Ritola KD, Sternson SM (2013). Parallel, redundant circuit organization for homeostatic control of feeding behavior. Cell.

[25] Meng F (2016). New inducible genetic method reveals critical roles of GABA in the control of feeding and metabolism. Proc. Natl Acad. Sci. USA.

[26] Patel HR (2006). Neuropeptide Y deficiency attenuates responses to fasting and high-fat diet in obesity-prone mice. Diabetes.

[27] Imai Y (2007). Insulin secretion is increased in pancreatic islets of neuropeptide Y-deficient mice. Endocrinology.

[28] Nguyen AD (2012). Y1 and Y5 receptors are both required for the regulation of food intake and energy homeostasis in mice. PLoS One.

[29] Hagan MM (2001). Immediate and prolonged patterns of Agouti-related peptide-(83-132)-induced c-Fos activation in hypothalamic and extrahypothalamic sites. Endocrinology.

[30] Tanida M, Shen J, Nagai K (2009). Possible role of the histaminergic system in autonomic and cardiovascular responses to neuropeptide Y. Neuropeptides.

[31] Shi YC (2013). Arcuate NPY controls sympathetic output and BAT function via a relay of tyrosine hydroxylase neurons in the PVN. Cell Metab..

[32] Marks JL, Waite K (1997). Intracerebroventricular neuropeptide Y acutely influences glucose metabolism and insulin sensitivity in the rat. J. Neuroendocrinol..

[33] Lin JY, Lin MZ, Steinbach P, Tsien RY (2009). Characterization of engineered channelrhodopsin variants with improved properties and kinetics. Biophys. J..

[34] Timper K (2018). Mild impairment of mitochondrial OXPHOS promotes fatty acid utilization in POMC neurons and improves glucose homeostasis in obesity. Cell Rep..

