# Supplementary material for: NPY mediates the rapid feeding and glucose metabolism regulatory functions of AgRP neurons
Source: Nat Commun. 2020 Jan 23;11:442. doi: 10.1038/s41467-020-14291-3 (PMC6978463; doi:10.1038/s41467-020-14291-3)
Supplement: Supplementary file 1 — Supplementary Information [file 41467_2020_14291_MOESM1_ESM.pdf]

Supplementary Information

NPY Mediates the Rapid Feeding and Glucose Metabolism Regulatory Functions of AgRP Neurons

Ruud, L.E., Pereira, M.M.A et. al

Supplementary Figure 1

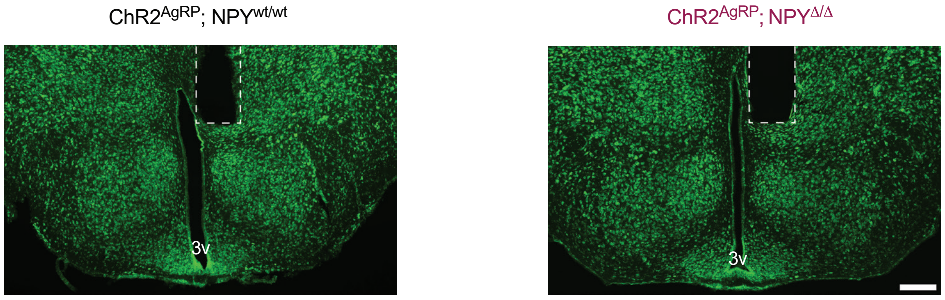

Representative images of fiber-optic cannula placements above the ARC in control mice (ChR2^{AgRP}; NPY$^{wt/wt}$) and in NPY-deficient mice (ChR2^{AgRP}; NPY$^{\Delta/\Delta}$). Scale bar = 200 μm. Related to Fig. 1 to Fig. 5.

Supplementary Figure 2

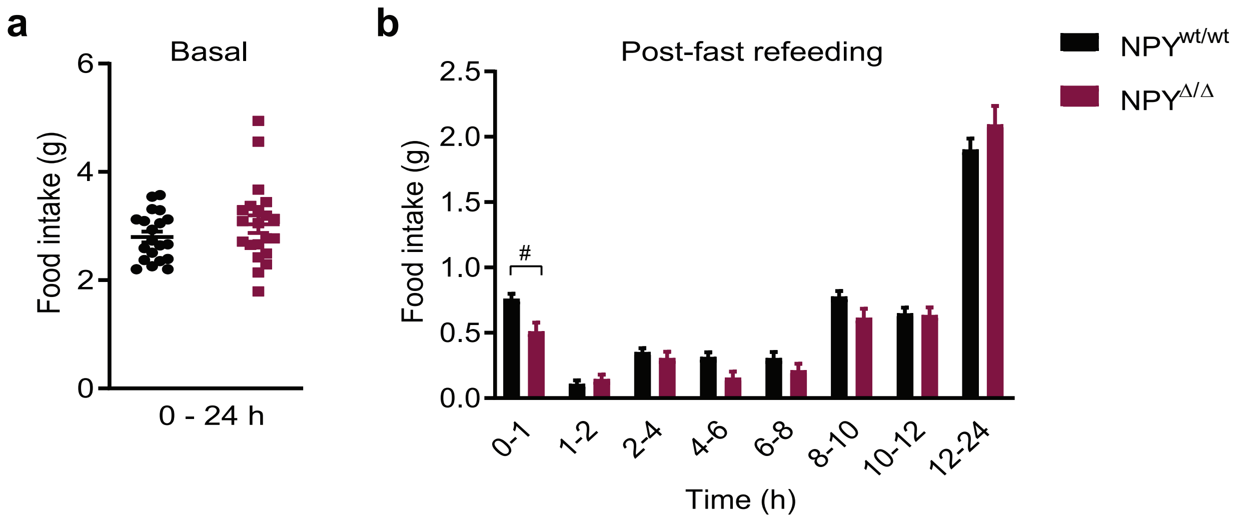

Food intake in NPY-deficient and control mice. a, $NPY^{wt/wt}$ and $NPY^{\Delta/\Delta}$ mice display similar basal food intake over 24 h (n = 20 mice for $NPY^{wt/wt}$ and n = 21 mice for $NPY^{\Delta/\Delta}$). **b,** The absence of NPY impairs rapid food intake in the first hour of re-feeding (n = 19 mice for $NPY^{wt/wt}$ and n = 14 mice for $NPY^{\Delta/\Delta}$). Data are shown as mean ± s.e.m. Statistical analysis is represented by $^{\#}p \leq 0.05$ as determined by two-way ANOVA followed by Sidak post hoc test. A Student's t-test was performed to analyse data shown in a.

Supplementary Figure 3

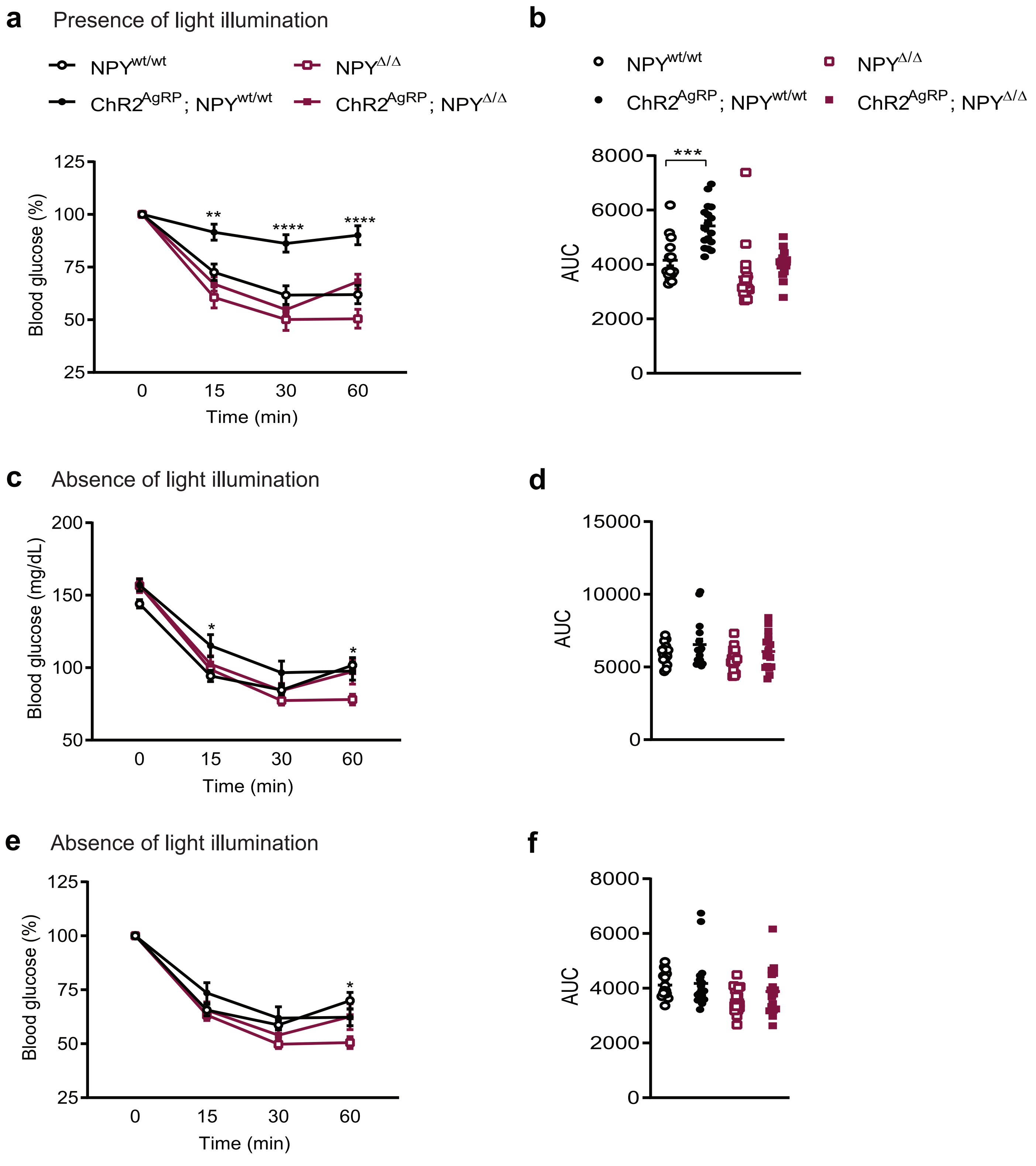

NPY is necessary for the acute insulin resistance upon optogenetic activation of AgRP neurons. a, b, Insulin tolerance test (ITT) in the presence and in the absence of NPY upon optogenetic activation of AgRP neurons. Values were normalized to blood glucose at the time of insulin injection (area under the curve (AUC); n = 15 mice for $NPY^{wt/wt}$, n = 17 mice $ChR2^{AgRP}$; $NPY^{wt/wt}$ and $ChR2^{AgRP}$; $NPY^{\Delta/\Delta}$ and n = 18 mice for $NPY^{\Delta/\Delta}$). **c, d,** Blood glucose levels in NPY-deficient and control mice during ITT in the absence of light illumination (area under the curve (AUC); n = 15 mice for $NPY^{wt/wt}$, n = 17 mice for $ChR2^{AgRP}$; $NPY^{wt/wt}$ and $ChR2^{AgRP}$; $NPY^{\Delta/\Delta}$ and n = 18 mice for $NPY^{\Delta/\Delta}$). **e, f,** Blood glucose levels in NPY-deficient and control mice during ITT in the absence of light illumination. Values were normalized to blood glucose at the time of insulin injection (area under the curve (AUC); n = 15 mice for $NPY^{wt/wt}$, n = 17 mice for $ChR2^{AgRP}$; $NPY^{wt/wt}$ and $ChR2^{AgRP}$; $NPY^{\Delta/\Delta}$ and n = 18 mice for $NPY^{\Delta/\Delta}$). Data are shown as mean ± s.e.m. Statistical analysis is represented by $^{*}p \le 0.05$, $^{**}p \le 0.01$, $^{***}p \le 0.001$ and $^{****}p \le 0.0001$ as determined by two-way ANOVA (panels a, c and e) or one-way ANOVA (panel b) followed by Tukey post hoc test. Related to Fig. 4.

Supplementary Figure 4

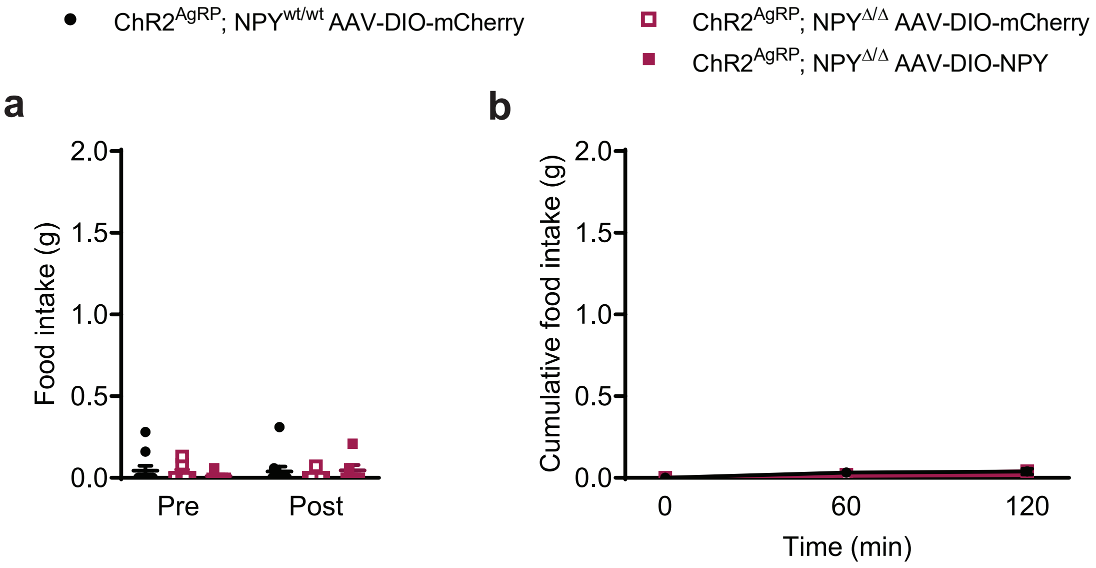

Control experiments for selective NPY re-expression in AgRP neurons. a, Food intake 1 h before (pre) and after (post) light illumination in mice injected with AAV-DIO-mCherry or AAV-DIO-NPY bilaterally in the ARC. **b,** Cumulative food intake in the absence of light illumination. n = 10 and n = 7 mice for $ChR2^{AgRP}$; $NPY^{wt/wt}$ and $ChR2^{AgRP}$; $NPY^{\Delta/\Delta}$ injected with mCherry virus and n = 6 mice for $ChR2^{AgRP}$; $NPY^{\Delta/\Delta}$ injected with NPY virus. Data are shown as mean ± s.e.m. Related to Fig. 5.

Supplementary Figure 5

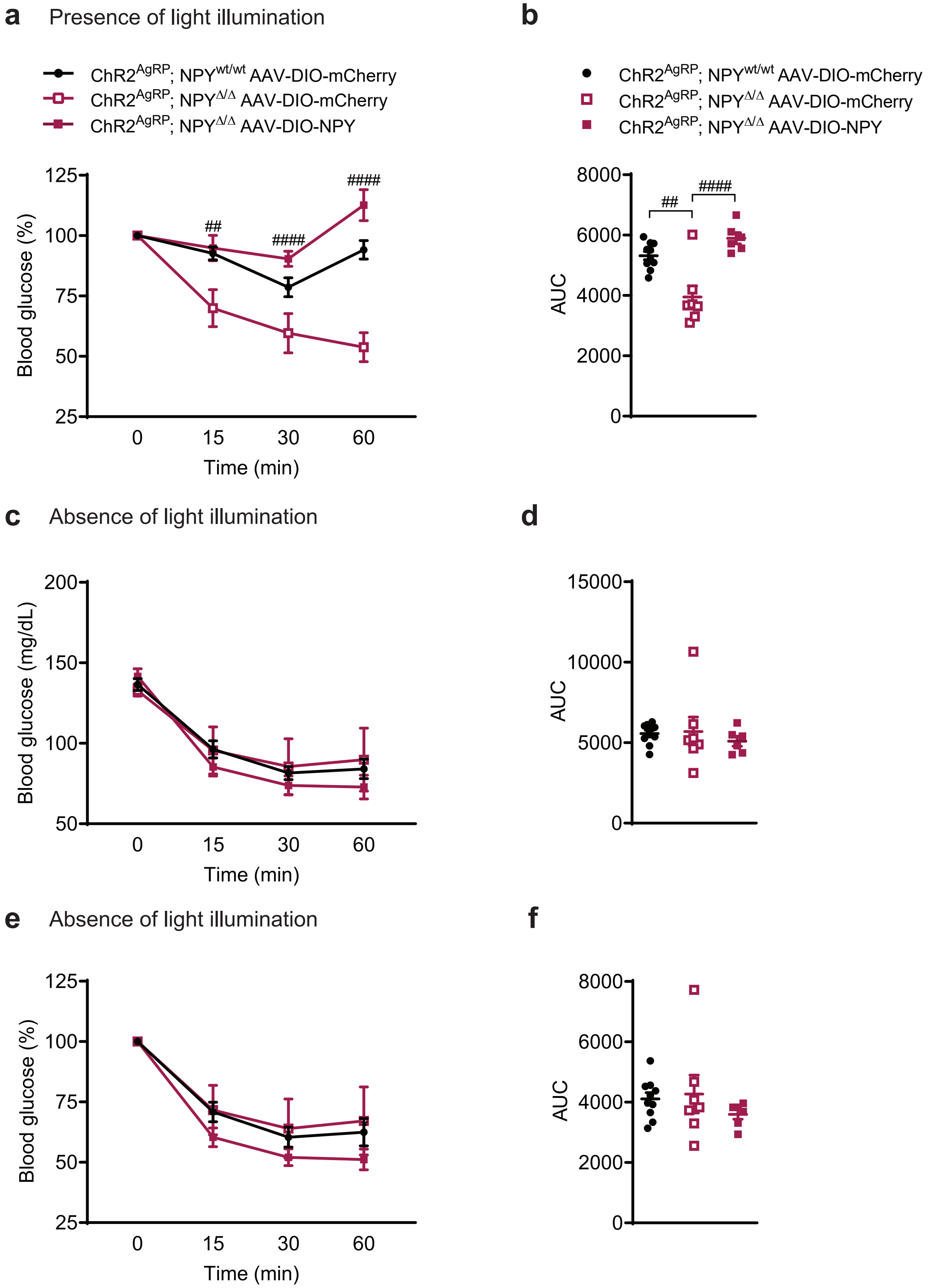

Selective re-expression of NPY in AgRP neurons. a, b, ITT and corresponding AUC during optogenetic AgRP neuronal stimulation in virally transduced NPY-deficient and wildtype control mice (mCherry) and in NPY-deficient mice re-expressing NPY in the ARC. Values were normalized to blood glucose at the time of insulin injection. **c, d,** ITT and corresponding AUC in the absence of light illumination in virally transduced NPY-deficient and wildtype control mice (mCherry) and in NPY-deficient mice re-expressing NPY in the ARC. **e, f,** ITT and corresponding AUC in the absence of light illumination in virally transduced NPY-deficient and wildtype control mice (mCherry) and in NPY-deficient mice re-expressing NPY in the ARC. Values were normalized to blood glucose at the time of insulin injection. n = 10 and n = 7 mice for ChR2^{AgRP}; NPY$^{wt/wt}$ and ChR2^{AgRP}; NPY$^{\Delta/\Delta}$ (mCherry virus) and n = 6 mice for ChR2^{AgRP}; NPY$^{\Delta/\Delta}$ (NPY virus). Data are shown as mean ± s.e.m. Statistical analysis is represented by $^{\#\#}p \leq 0.01$, $^{\#\#\#\#}p \leq 0.0001$ as determined by two-way ANOVA (panels a, c and e) or one-way ANOVA (panel b) followed by Tukey post hoc test. Related to Fig. 5.

Supplementary Figure 6

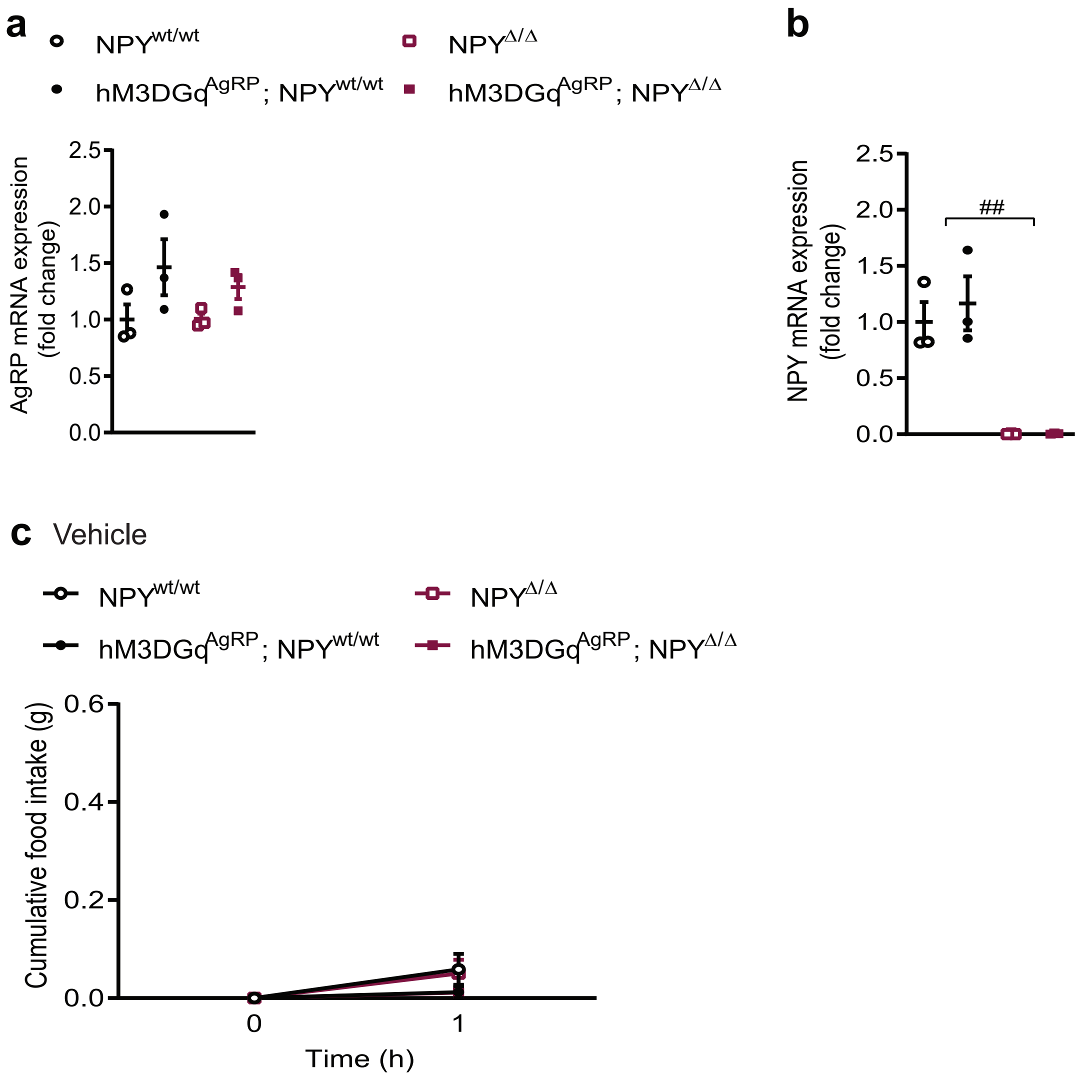

Control experiments for the chemogenetics approach. a, AgRP mRNA levels, as measured with qPCR, are unaltered in NPY$^{\Delta/\Delta}$ mice upon chemogenetic activation of AgRP neurons (n = 3 mice per group, all animals received an ip CNO injection). **b,** NPY expression is absent in NPY$^{\Delta/\Delta}$ mice (n = 3 mice per group, all groups were injected with ip CNO). **c,** Food intake upon ip vehicle injection in the presence and in the absence of NPY (n = 8 mice for NPY$^{wt/wt}$, n = 5 for hM3DGq^{AgRP}; NPY$^{wt/wt}$, n = 9 for NPY$^{\Delta/\Delta}$ and n = 7 mice for hM3DGq^{AgRP}; NPY$^{\Delta/\Delta}$). Data are shown as mean ± s.e.m. Statistical analysis is represented by $^{\#\#}p \leq 0.01$ as determined by one-way ANOVA followed by Tukey post hoc test. Related to Fig. 6.

Supplementary Figure 7

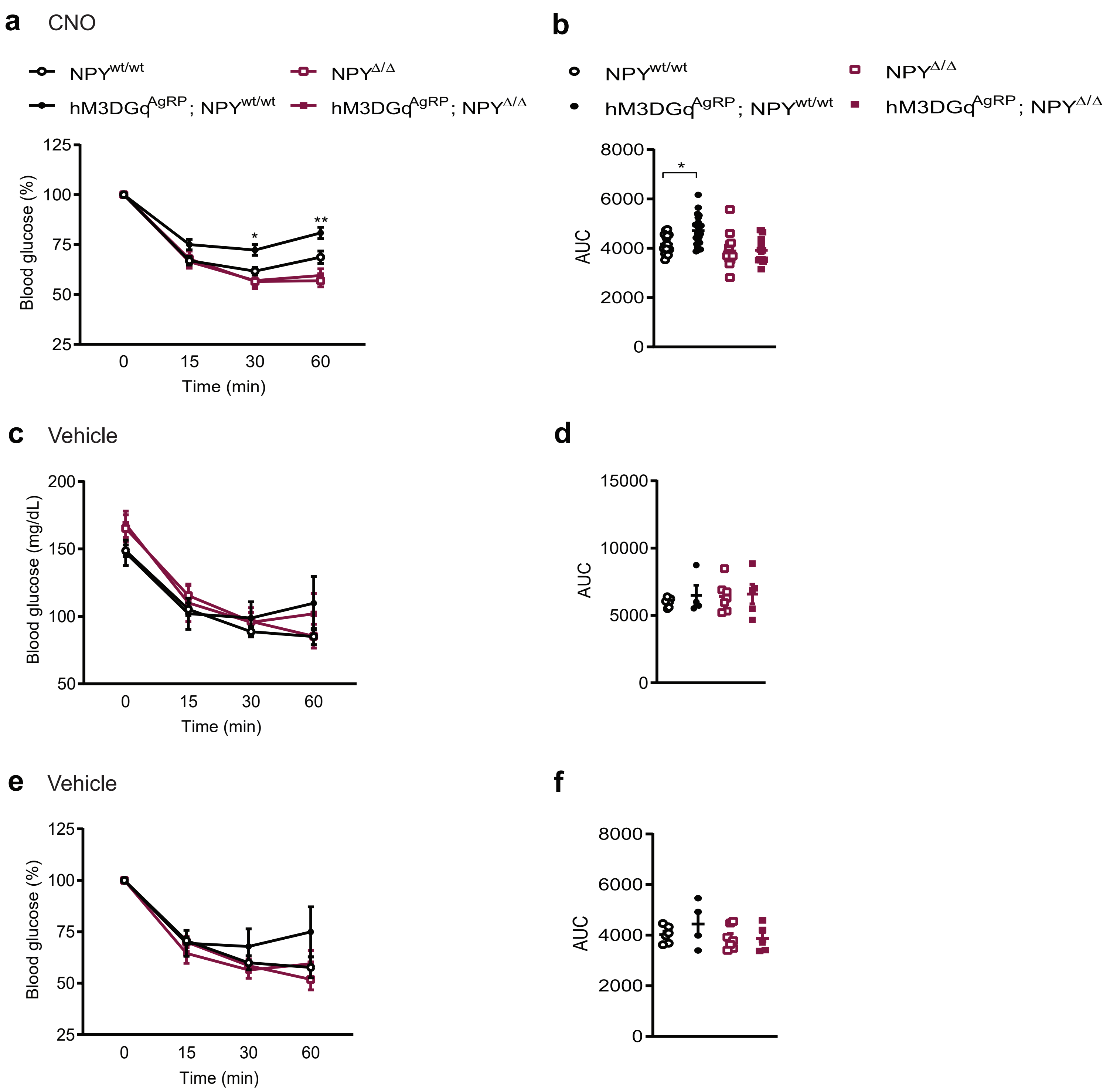

NPY is necessary for the acute insulin resistance upon chemogenetic activation of AgRP neurons. a, b, ITT in NPY-expressing and NPY-deficient mice upon chemogenetic activation of AgRP neurons with values normalized to blood glucose at the time of insulin injection (area under the curve (AUC); n = 17 mice for $NPY^{wt/wt}$, n = 23 mice for $hM3DGq^{AgRP}$; $NPY^{wt/wt}$, n = 14 mice for $NPY^{\Delta/\Delta}$ and n = 15 mice for $hM3DGq^{AgRP}$; $NPY^{\Delta/\Delta}$, all animals received an ip CNO injection. **c, d,** ITT in NPY-expressing and NPY-deficient mice upon ip vehicle injection (area under the curve (AUC); n = 6 mice for $NPY^{wt/wt}$, n = 4 mice for $hM3DGq^{AgRP}$; $NPY^{wt/wt}$, n = 7 for $NPY^{\Delta/\Delta}$ and n = 5 mice for $hM3DGq^{AgRP}$; $NPY^{\Delta/\Delta}$). **e, f,** ITT in NPY-expressing and NPY-deficient mice upon vehicle ip injection with values normalized to blood glucose at the time of insulin injection (area under the curve (AUC); n = 6 mice for $NPY^{wt/wt}$, n = 4 mice for $hM3DGq^{AgRP}$; $NPY^{wt/wt}$, n = 7 for $NPY^{\Delta/\Delta}$ and n = 5 mice for $hM3DGq^{AgRP}$; $NPY^{\Delta/\Delta}$). Data are shown as mean ± s.e.m. Statistical analysis is represented by *$p \leq 0.05$ and **$p \leq 0.01$ as determined by two-way ANOVA (panel a) or one-way ANOVA (panel b) followed by Tukey post hoc test. Related to Fig. 7.
